# Innate Immune Responses and Antioxidant/Oxidant Imbalance Are Major Determinants of Human Chagas Disease

**DOI:** 10.1371/journal.pntd.0002364

**Published:** 2013-08-08

**Authors:** Monisha Dhiman, Yun A. Coronado, Cecilia K. Vallejo, John R. Petersen, Adetoun Ejilemele, Sonia Nuñez, Maria Paola Zago, Heidi Spratt, Nisha Jain Garg

**Affiliations:** 1 Department of Microbiology and Immunology, School of Medicine, University of Texas Medical Branch (UTMB), Galveston, Texas, United States of America; 2 Department of Pathology, University of Texas Medical Branch, Galveston, Texas, United States of America; 3 Hospital Público de Gestión Descentralizada San Bernardo (HPGDSA), Salta, Argentina; 4 Instituto de Patología Experimental (IPE), Universidad Nacional de Salta (UNSa), Salta, Argentina; 5 Departments of Biochemistry and Molecular Biology, and Preventive Medicine and Community Health, University of Texas Medical Branch, Galveston, Texas, United States of America; 6 Faculty of the Center for Tropical Diseases, and the Institute for Human Infections and Immunity, University of Texas Medical Branch, Galveston, Texas, United States of America; Institute of Tropical Medicine (NEKKEN), Japan

## Abstract

**Background:**

We investigated the pathological and diagnostic role of selected markers of inflammation, oxidant/antioxidant status, and cellular injury in human Chagas disease.

**Methods:**

Seropositive/chagasic subjects characterized as clinically-symptomatic or clinically-asymptomatic (n = 116), seronegative/cardiac subjects (n = 102), and seronegative/healthy subjects (n = 45) were analyzed for peripheral blood biomarkers.

**Results:**

Seropositive/chagasic subjects exhibited an increase in sera or plasma levels of myeloperoxidase (MPO, 2.8-fold), advanced oxidation protein products (AOPP, 56%), nitrite (5.7-fold), lipid peroxides (LPO, 12–17-fold) and malondialdehyde (MDA, 4–6-fold); and a decline in superoxide dismutase (SOD, 52%) and glutathione (GSH, 75%) contents. Correlation analysis identified a significant (p<0.001) linear relationship between inflammatory markers (AOPP/nitrite: r = 0.877), inflammation and antioxidant/oxidant status (AOPP/glutathione peroxidase (GPX): r = 0.902, AOPP/GSH: r = 0.806, Nitrite/GPX: 0.773, Nitrite/LPO: 0.805, MDA/MPO: 0.718), and antioxidant/oxidant levels (GPX/MDA: r = 0.768) in chagasic subjects. Of these, MPO, LPO and nitrite biomarkers were highly specific and sensitive for distinguishing seropositive/chagasic subjects from seronegative/healthy controls (p<0.001, training and fitting AUC/ROC >0.95). The MPO (r = 0.664) and LPO (r = 0.841) levels were also correlated with clinical disease state in chagasic subjects (p<0.001). Seronegative/cardiac subjects exhibited up to 77% decline in SOD, 3–5-fold increase in LPO and glutamate pyruvate transaminase (GPT) levels, and statistically insignificant change in MPO, AOPP, MDA, GPX, GSH, and creatine kinase (CK) levels.

**Conclusions:**

The interlinked effects of innate immune responses and antioxidant/oxidant imbalance are major determinants of human Chagas disease. The MPO, LPO and nitrite are excellent biomarkers for diagnosing seropositive/chagasic subjects, and MPO and LPO levels have potential utility in identifying clinical severity of Chagas disease.

## Author Summary

Chagas disease is a chronic disease of the heart, and caused by *Trypanosoma cruzi* infection. In this study, we have monitored the biomarkers of inflammation caused by innate immune cells, oxidative stress, and antioxidant status in seropositive/chagasic, seronegative/cardiac disease, and seronegative/healthy subjects. Our goal was to evaluate the diagnostic efficacy of selected biomarkers, and determine if any of the biomarkers are good indicators of clinical severity of Chagas disease. We also determined whether sera or plasma serve as a better source, and if sample storage affects the estimation of the selected biomarkers. Our data suggest that innate immune responses and antioxidant/oxidant imbalance are inter-linked pathological events in Chagas disease. We have identified peripheral blood markers (myeloperoxidase, lipid hydroperoxides and nitrite) that strongly distinguish seropositive/chagasic subjects from controls. Further, we found that myeloperoxidase and lipid hydroperoxide levels have potential utility in identifying seropositive subjects at risk of developing clinically symptomatic disease.

## Introduction

*Trypanosoma cruzi (Tc)* is the etiologic agent of Chagas disease. Acute infection of *Tc* is clinically unapparent and silent. Most (>95%) acutely-infected individuals enter a clinically-asymptomatic phase defined by positive serological and parasitological tests, and the absence of cardiac abnormalities [1]. Approximately 30% of the infected individuals, several years after initial exposure, develop clinically-symptomatic disease with evidence of cardiomegaly, ventricular dilation and arrhythmia, leading to heart failure [2].

Because acute *Tc* infection is not clinically apparent, it is difficult to study the role of innate immune cells in mediating parasite control in human infection. A few studies have suggested that macrophages, neutrophils and natural killer cells control parasite replication in the early stages of human infection [3]–[4]. Experimental studies indicate that *Tc*-derived molecules engage toll-like receptors to drive activation of macrophages and neutrophils that then produce oxidative burst [5], nitric oxide (^•^NO), and HOCl supported by activation of NADPH oxidase, inducible nitric oxide synthase (iNOS) [6]–[7], and MPO [8], respectively. Depletion of neutrophils in murine models of infection exacerbated disease associated with splenic decline in expression of Th1 cytokines [9]. Thus, the significance of innate immune cells in control of *Tc*-infection is recognized. Others have noted infiltration of neutrophils and macrophages in chronic myocardium [10]–[12], though their role in Chagas disease is not clear.

Mitochondrial oxidative dysfunction resulting in increased release of electrons to O_2_ serves as a main source of superoxide generation and oxidative stress in chagasic myocardium [13]–[14]. The byproducts of reactive oxygen species (ROS) and reactive nitrogen species (RNS) are highly stable, and can cause oxidation of proteins, lipids, and DNA, leading to deterioration of cellular structure and function. The sustained oxidative damage, evidenced by consistent increase in myocardial protein carbonylation and MDA contents [15]–[16], in chagasic hearts occurred not only due to increased free radical generation, but was also exacerbated by inefficient antioxidant capacity [17].

In this study, our primary objective was to investigate the diagnostic efficacy of the markers of inflammation, oxidative stress, and antioxidant status in identifying *Tc*-infection and severity of Chagas disease. For this, we evaluated the oxidative biomarkers (MDA, LPO), inflammatory mediators (MPO, AOPP, and nitrite); and antioxidants (GSH, SOD, GPX) in sera and plasma of seronegative/healthy controls and seropositive/chagasic subjects. Further, we examined the GPT and CK activities as biomarkers of cellular injury. Our second objective was to determine whether sera or plasma serve as a better source, and if sample storage affects the estimation of the selected biomarkers. Finally, statistical analysis was performed to identify the correlation between biomarkers and/or clinical disease state in seropositive/chagasic subjects. We analyzed plasma samples from seronegative/cardiac subjects to determine if the selected biomarkers are specific to chagasic disease or are general indicators of cardiac involvement.

## Materials and Methods

### Human samples

All procedures were approved by the Institutional Review Boards at UTMB, Galveston and Universidad Nacional de Salta (UNSa), Argentina. Human sera samples used in this study were obtained from Salta Argentina (located at the border of Bolivia) known to be endemic for *T. cruzi* transmission and human infection. Sera samples from seronegative, healthy individuals and seronegative individuals exhibiting cardiac disease of other etiologies were obtained from the same geographical area in Argentina as well as from UTMB clinics, and were used as controls. All samples were decoded and de-identified before they were provided for research purposes. Written informed consent was obtained from all individuals. Subjects with co-morbid diseases, e.g., HIV/AIDS, *Leishmaniasis*, autoimmune disorders, or chronic hepatic, renal or pulmonary disease were excluded. Patients' detailed information is presented in Table 1.

**Table 1:** Characterization of the subjects included in the study.

Clinical characterization	Enrolled subjects (numbers)	Age in years (mean ± SD)	Sex Males (%)
**Salta, Argentina** a			
*Seropositive for T. cruzi-specific antibodies*			
Chagasic 0	n = 54	51.3±7.6	47 (40%)
Chagasic 1	n = 30	47.6±12	15 (50%)
Chagasic 2	n = 17	53.6±12.9	10 (55%)
Chagasic 3	n = 15	52.3±8.2	5 (33%)
*Seronegative for T. cruzi-specific antibodies*			
Healthy, no disease	n = 20	35±16.2	9 (45%)
**Non-endemic area** b			
Seronegative, healthy, no disease	n = 25	39±14.7	10 (40%)
Seronegative, other Cardiomyopathy	n = 102	59±18.2	61 (60%)

a Subjects were screened for *T. cruzi*-specific antibodies by Wiener Chagatest-ELISA and Wiener Chagatest-HAI kits. Clinical exam included physical exam, electrocardiography and echocardiography.

b Seronegative subjects from non-endemic areas were screened for *T. cruzi*-specific antibody response. Seronegative/cardiac patients exhibiting clinical symptoms were identified based upon blood levels of NT-proBNP to be >2000 pg/ml (normal <450 pg/ml).

Blood samples were collected with K_3_EDTA (1.5-mg/ml blood) or without anticoagulant to obtain plasma and serum, respectively. *Tc*-specific antibodies in sera samples were monitored by an enzyme-linked immunosorbent assay using a Wiener Chagatest-ELISA recombinant v.4.0 kit comprising six recombinant proteins known to be expressed in mammalian stage of *T. cruzi* in isolates circulating in Latin America. Briefly, 96-well plates were coated with recombinant proteins, and then sequentially incubated with 20-µl sera samples (1∶20 dilution), HRP-conjugated human monoclonal anti-IgG, and color developed with chromogenic substrate monitored by spectrometry at 450 nm (cut-off value: average of seronegative samples (<0.1 O.D.)+0.2 O.D., i.e. ≥0.3). Serological tests were also done following the specifications of the commercial IHA test kit (Wiener Chagatest-HAI). Briefly, sera samples (25-µl 4-fold dilutions) were mixed with red blood cells sensitized with *T. cruzi* cytoplasmic and membrane antigens, and agglutination monitored. The titer was defined as the highest serum dilution presenting agglutination (positive ≥1∶16 dilution). Those positive by both tests were identified as seropositive [18].

Cardiologists with >30 years of cumulative experience in diagnosis and treatment of cardiomyopathy patients of all etiologies performed the clinical characterization. Clinical data included medical history, physical examination and subjective complaint of frequency and severity of exertional dyspnea. Electrocardiography (12-lead at rest and 3-lead with exercise) was obtained to identify heart rate variability, ventricular arrhythmia, atrial- and ventricular-conduction defects, bundle branch block, and S-T segment and T wave changes. Transthoracic echocardiography is one of the most important diagnostic procedures to obtain objective information regarding the left ventricular (LV) contractile function. The following routine 2D and Doppler echo were evaluated: 1) end systolic and end-diastolic LV dimensions, 2) mitral inflow pattern, and 3) pulmonary vein flow pattern. LV ejection fraction (LVEF) was used to represent LV systolic function. LV diastolic function was assessed based on Doppler mitral inflow patterns and Doppler pulmonary venous flow patterns, and the severity of diastolic dysfunction graded as I–IV [19]. Global/regional wall-motion abnormalities and inter-cavity thrombus were also recorded. Seronegative/healthy subjects (n = 45) exhibiting no history or clinical symptoms of heart disease were used as controls. Seropositive/chagasic patients (n = 116) were classified based on clinical exam as follows: CD0: no echocardiography abnormalities, no left ventricular dilatations, and ≥70% ejection fraction (EF) indicating preserved systolic function, CD1: negligible to minor EKG alterations, EF: 55–70%, no indication of heart involvement; CD2: a degree of heart involvement with systolic dysfunction (EF: 40–55%); and CD3: moderate to severe systolic dysfunction (EF ≤40%), left ventricular dilatation (diastolic diameter ≥57 mm), and/or potential signs of congestive heart failure. For the statistical analysis purpose, chagasic subjects in CD0–CD1 and CD2–CD3 clinical state were classified as clinically asymptomatic and clinically symptomatic, respectively. Seronegative subjects with ischemic or non-ischemic cardiovascular involvement (n = 102) were identified based upon clinical exam, and blood levels of NT-proBNP >2000 ng/ml that reflects NYHA classification II–III of cardiac involvement (similar to CD2–CD3 chagasic group). All assays were conducted in 96-well format.

### Inflammatory markers

MPO activity was determined by a dianisidine-H_2_O_2_ method [20]. Briefly, samples (10 µl) were added to 0.53 mM o-dianisidine dihydrochloride (Sigma-Aldrich) and 0.15 mM H_2_O_2_ in 50 mM K_2_HPO_4_ buffer (pH 6.0), and absorbance recorded at 460 nm on a SpectraMax 190 microplate reader (Molecular Devices). Sample protein content was measured by Bradford method, and 1 unit MPO was defined as that degrading 1 nmol H_2_O_2_/min at 25°C (ε = 11300 M^−1^.cm^−1^).

Dityrosine-containing protein cross-linking products, designated as AOPP, are the products of HOCl-induced chlorination of amines. Samples (20 µl/well) were mixed with 10 µl of 1.16 M KI and 20 µl of 100% acetic acid (final volume: 200 µl). Absorbance was recorded at 340 nm, and AOPP concentration expressed as chloramine-T equivalents (standard curve: 0–100 µmol chloramine-T/ml) [21].

The ^•^NO level (indicator of iNOS activity), was monitored by using Nitrate/nitrite Assay Kit (Cayman). Briefly, samples (10 µg protein) were reduced with 0.01 unit/100 µl of nitrite reductase, and incubated for 10 min with 100 µl of 1% sulfanilamide made in 5% phosphoric acid/0.1% N-(1-napthyl) ethylenediamine dihydrochloride (1∶1,v/v). Formation of diazonium salt was monitored at 545 nm (standard curve: 2–50 µM sodium nitrite).

### Oxidative stress markers

We utilized LPO Assay Kit (Cayman) to measure the lipid peroxides. Peripheral blood LPOs were extracted into chloroform, and added to 50 µl of 4.5 mM FeSO_4_ in 0.2M HCl/3% NH_4_SCN in methanol (1∶1, v/v). The redox reaction with ferrous ions was stopped after 5-min, and absorbance monitored at 500 nm (standard curve: 0–500-µM 13-hydroperoxy octadecadienoic acid).

MDA level was measured by using the QuantiChrom TBARS Assay Kit (BioAssay Systems). Samples (10 µl) were suspended in 200 µl thiobarbituric acid (TBA) reagent and heated at 100°C for 60 min. After cooling, the reaction mixture was centrifuged, and TBARS equivalent in supernatants monitored at 535 nm (standard curve: 0–30 µM MDA).

### Antioxidants

To measure SOD activity, we utilized SOD Assay Kit (Cayman). Briefly samples (10 µl) were mixed with 50 µl of reaction mixture), reaction was initiated with xanthine oxidase, and the reduction of NBT by O_2_^•−^ monitored at 450 nm [22]. One unit of SOD activity was defined as that producing 50% dismutation of O_2_^•−^ radical.

To measure GPX activity, samples (10 µl) were added to 90 µl assay buffer (50 mM Tris HCl, 0.5 mM EDTA, pH 7.6) containing, 2 mM GSH and 100-µU glutathione reductase (GSR) and 0.15 mM NADPH. GPX catalyzed reduction of cumene hydroperoxide (0.8 mM) coupled with GSR-dependent NADPH oxidation was recorded at 340 nm (ε = 0.00373 µM^−1^) [23].

For GSH content, 100 µl of the supernatants from the deproteinated samples were added to 200 µl of assay buffer (0.1 M sodium phosphate, 5 mM EDTA buffer, pH 7.5) containing 0.1M triethanolamine, 0.6 mM 5-5′-dithiobis 2-nitrobenzoic acid (DTNB) and 0.15 mM NADPH. The reaction was initiated with 10 µl GSR (3 U/ml), and the reduction of DTNB by GSH monitored at 412 nm (standard curve: 0–8 µM GSSG).

### Cellular injury markers

GPT catalyzes reversible transamination between alanine and α-ketoglutarate to form pyruvate and glutamate. Briefly, 5 µl samples were added to 100 µl assay buffer (100 mM Tris HCl pH 7.8, 100 mM NaHCO_3_, 0.1 mM pyridoxal 5-phosphate, 0.01% sodium azide) containing L-alanine, NADH and lactate dehydrogenase (LDH). The reaction was initiated with addition of 150 mM α-ketoglutarate, and NADH oxidation was recorded at 340 nm. One unit of GPT converted 1 µmol of α-ketoglutarate to L-glutamate per minute at 37°C (ε = 4.11 mM^−1^).

Creatine kinase activity was determined using EnzyChrom CK Assay Kit (BioAssay Systems). Briefly, samples (5 µl) were mixed with 100 µl assay buffer, and CK-dependent formation of ATP was coupled with glucose (20 µM) phosphorylation by hexokinase (250 units) and subsequent oxidation of glucose-6-phosphate by NADP (2 µM) in presence of glucose-6-phosphate dehydrogenase, the resultant NADPH monitored at 340 nm. One unit of CK activity transferred 1 µmol phosphate/min at pH 6.0 (detection limit: 5-nU/µl).

### Data analysis

All samples were analyzed in triplicate, and data are presented as mean±S.D. All data were analyzed using SigmaPlot v12. Data were log transformed and when normally distributed, analyzed by Student *t* test (comparison of 2-groups) and 1-way analysis of variance (ANOVA) with Tukey's post-hoc test (comparison of multiple groups). When the data were not normally distributed, non-parametric tests Mann-Whitney (M-W, comparison of two groups) and Kruskal-Wallis with Dunn's method (comparison of multiple groups) were employed. Significance was accepted at p<0.05. Pearson's (normally distributed data variables) and Spearman's (non-normally distributed data variables) correlation analysis were performed to determine the strength of linear relationship between different parameters. Multivariate Adaptive Regression Splines (MARS) was employed to model the utility of inter-relational changes in multiple variables in distinguishing infection and disease status [24]. The sensitivity and specificity of the biomarkers was validated by receiver operator characteristics (ROC) curves.

## Results

### Inflammatory markers: Sera levels of MPO and plasma levels of AOPP and nitrite are enhanced in seropositive/chagasic subjects

**Figure 1:**
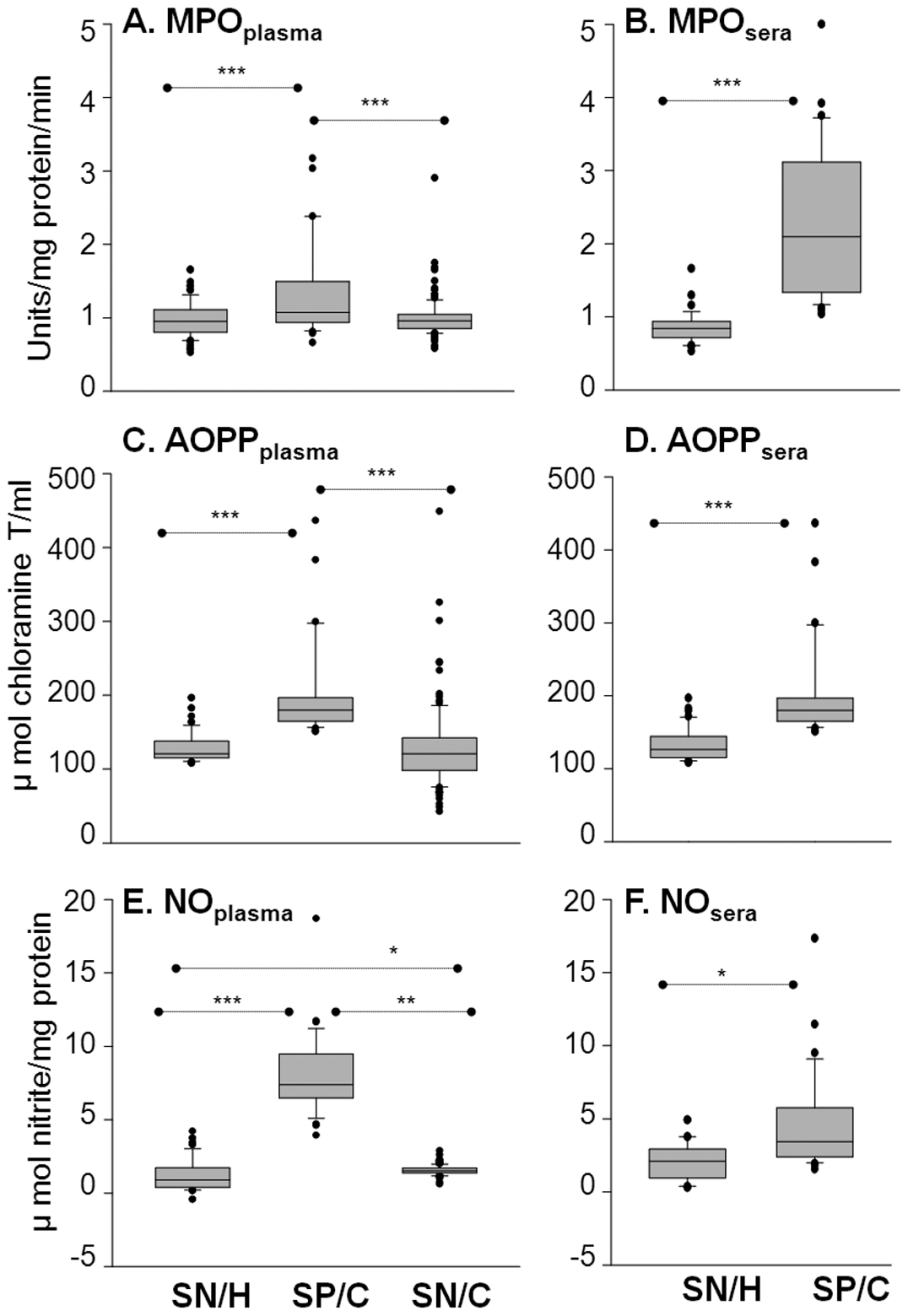
Neutrophil (MPO) and macrophage (iNOS) activation indicate inflammatory state of seropositive/chagasic subjects. Plasma and sera samples were obtained from seronegative/healthy (SN/H), seropositive/chagasic (SP/C) and seronegative/cardiac (SN/C) subjects, as described in Materials and Methods. Shown are the plasma *(* ***A,C,E*** *)* and sera *(* ***B,D,F*** *)* levels of MPO activity (**A&B**), AOPP contents (**C&D**) and nitrite levels (**E&F**). For all figures, data (mean of triplicate observations from each sample) are presented as box plot. The horizontal lines of the box (bottom to top) depict the lower quartile (Q1, cuts off lowest 25% of the data), median (Q2, middle value), and upper quartile (Q3, cuts off the highest 25% of the data). The lower and upper whiskers depict the smallest and largest non-outlier observations, respectively, and solid dots represent the outliers. The spacing between the different parts of the box indicates the degree of dispersion (spread). Standard deviation for triplicate observations for all samples was <12%. Significance is shown as *p<0.05, **p<0.05, and ***p<0.001.

Plasma and sera samples from enrolled subjects were stored at −80°C, and thawed when utilized. Summarized data (mean ± SD and range values) are presented in Table S1. MPO activity, a marker of neutrophil activation, was increased by 32% and 2.8-fold in plasma and sera, respectively, of seropositive/chagasic subjects as compared to that noted in seronegative/healthy and seronegative/cardiac subjects (*p*<0.001, Fig. 1A&B). All seropositive subjects exhibited sera MPO activity above the mean_seronegative_ level, while none of the seronegative/healthy controls exhibited MPO above the mean_seropositive_ level. MPO activity was not significantly altered in seronegative/cardiac subjects (Fig. 1A).

AOPPs are secondary marker of MPO activation. AOPP contents were increased by 56% and 26% in plasma and sera, respectively, of seropositive/chagasic subjects as compared to that noted in seronegative/healthy and seronegative/cardiac subjects (*p*<0.001, Fig. 1C&D). All seropositive subjects exhibited plasma AOPP contents above the mean_seronegative_ level, while only 2% seronegative/healthy subjects exhibited AOPP contents above the mean_seropositive_ level. AOPP contents were not significantly altered in plasma of seronegative/cardiac subjects (Fig. 1C).

Activated macrophages produce iNOS-mediated ^•^NO that is then reduced to nitrite. Seropositive/chagasic subjects exhibited a 5.8-fold and 5.2-fold increase in plasma levels of nitrite content when compared to that noted in plasma of seronegative/healthy and seronegative/cardiac subjects, respectively (*p*<0.001, Fig. 1E). Sera level of nitrite content was increased by 39% in seropositive/chagasic subjects (p<0.05, Fig. 1F). All seropositive subjects exhibited plasma nitrite contents above the mean_seronegative_ level while none of the seronegative/healthy subjects exhibited plasma nitrite contents above the mean_seropositive_ level. Plasma nitrite contents were marginally increased in seronegative/cardiac subjects when compared to the seronegative/healthy controls (Fig. 1E). Together, the data presented in Fig. 1 suggested that a) neutrophil (MPO) and macrophage (iNOS) activation contributed to inflammatory state in seropositive/chagasic subjects, and MPO activation resulted in the formation of deleterious oxidants. Sera were useful for monitoring the MPO activity, and plasma for analyzing AOPP level and iNOS activity. The seronegative/cardiac subjects with cardiac involvement of other etiologies exhibited very low to no changes in inflammatory markers.

### LPO and MDA are indicators of oxidative stress in Chagas disease

**Figure 2:**
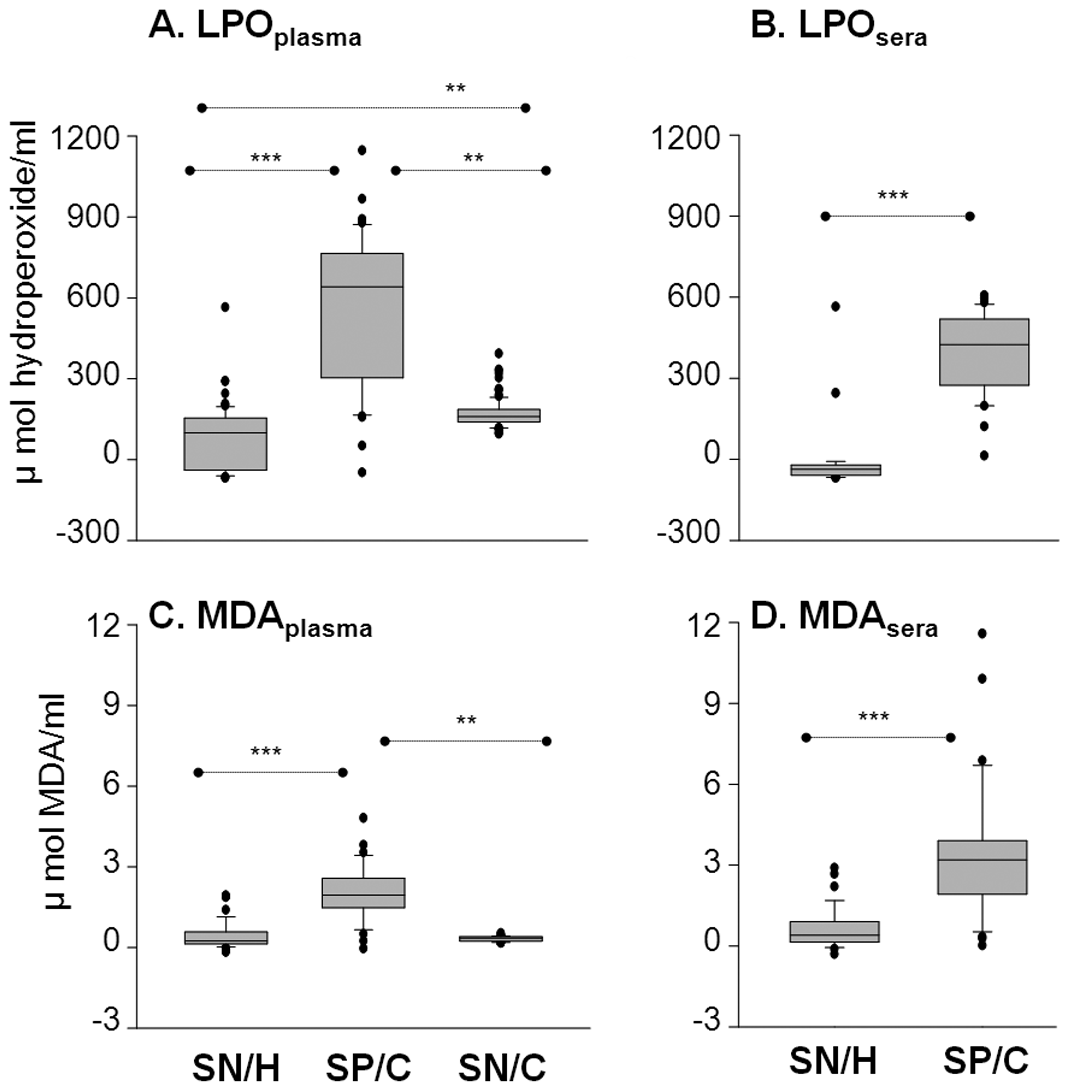
LPO and MDA are biomarkers of increased oxidative stress in chagasic subjects. The plasma *(* ***A&C*** *)* and sera *(* ***B&D*** *)* levels of lipid hydroperoxides (**A&B**) and malondialdehyde (**C&D**) were measured by spectrophotometry.

LPO refers to highly reactive hydroperoxides of saturated and unsaturated lipids, formed by oxidation. Plasma and sera levels of LPO were increased by 17-fold and 11.7-fold, respectively, in seropositive/chagasic subjects as compared to the seronegative/healthy controls (Fig. 2A&B, p<0.001). More than 97% of seropositive subjects had LPO level above the mean_seronegative_ level, while <2% seronegative/healthy subjects exhibited LPO above the mean_seropositive_ level. Seronegative/cardiac subjects also exhibited an increase in plasma LPO levels (up to 5-fold) when compared to the seronegative/healthy controls (Fig. 2A, p<0.01); however, the extent of increase in plasma LPO in seronegative/cardiac subjects was significantly lower than that noted in seropositive/chagasic subjects (Fig. 2A, p<0.01).

MDA are most stable breakdown products of LPO. Seropositive/chagasic subjects exhibited a 3.7-fold and 6.2-fold increase in plasma levels of MDA when compared to that noted in plasma of seronegative/healthy and seronegative/cardiac subjects, respectively (*p*<0.01-0.001, Fig. 2C). Sera level of MDA content was increased by 6-fold in seropositive/chagasic subjects (p<0.001, Fig. 2D). More than 91% of seropositive/chagasic subjects exhibited plasma and sera MDA levels above the mean_seronegative_ level, while none of the seronegative/healthy subjects exhibited MDA contents above the mean_seropositive_ level. The MDA level was not significantly changed in seronegative/cardiac subjects (Fig. 2C). These data showed that both sera and plasma are good source of samples for monitoring LPO and MDA levels, and oxidative stress induced damage is significantly increased in seropositive chagasic subjects.

### Antioxidants: Plasma levels of SOD and GSH are decreased in chagasic subjects

**Figure 3:**
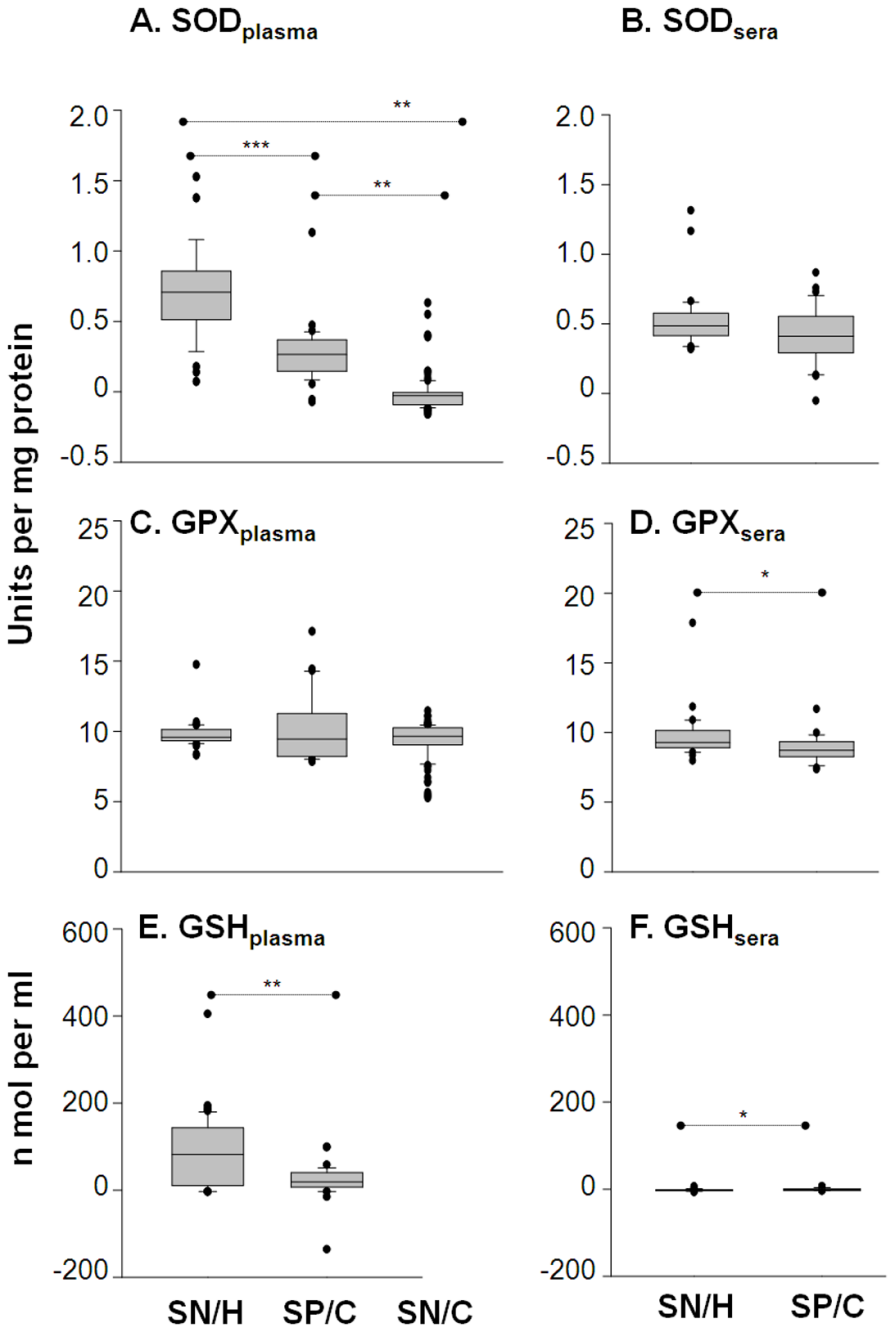
SOD and GSH are indicators of compromised antioxidant status in chagasic subjects. Plasma *(* ***A, C, E*** *)* and sera *(* ***B, D, F*** *)* levels of activities of the superoxide dismutase (**A&B**) and glutathione peroxidase (**C&D**) were determined by spectrophotometry. The glutathione contents (**E&F**) were measured by GSSG-DTNB recycling assay.

We examined the activities of SOD and GPX enzymes and GSH content, these being the important members of the antioxidant defense system. SOD catalyzes dismutation of O_2_^•−^ to H_2_O_2_ which is reduced to H_2_O and O_2_ by GPX using GSH. The plasma levels of SOD activity was decreased by >2-fold in seropositive/chagasic and seronegative/cardiac subjects when compared to that noted in seronegative/healthy controls (Fig. 3A, *p*<0.01-0.001). More than 97% of seropositive subjects exhibited plasma SOD activity below the mean_seronegative_ level. The plasma levels of GPX activity was not significantly different among seropositive/chagasic, seronegative/healthy and seronegative/cardiac groups (Fig. 3C); however, GSH level was affected in seropositive subjects. We noted the plasma levels of GSH were decreased by 4-fold in seropositive/chagasic subjects (Fig. 3E, *p*<0.001). More than 94% of the seropositive subjects exhibited plasma GSH levels at or below the mean_seronegative_ level, while <20% of the seronegative/healthy subjects exhibited plasma GSH levels below the mean_seropositive_ level. No significant difference in sera levels of SOD, GPX and GSH was observed in seropositive versus seronegative groups (Figs. 3B, 3D, 3F). These data suggest that peripheral antioxidant capacity, measured by plasma levels of SOD activity and GSH contents, is compromised in chagasic subjects. The seronegative/cardiac subjects exhibited a significant decline in plasma levels of SOD activity; however, this decline in SOD had no effect on overall antioxidant status, evidenced by no change in GPX and GSH levels.

### CK and GPT are not good indicators of tissue injury in Chagas disease

**Figure 4:**
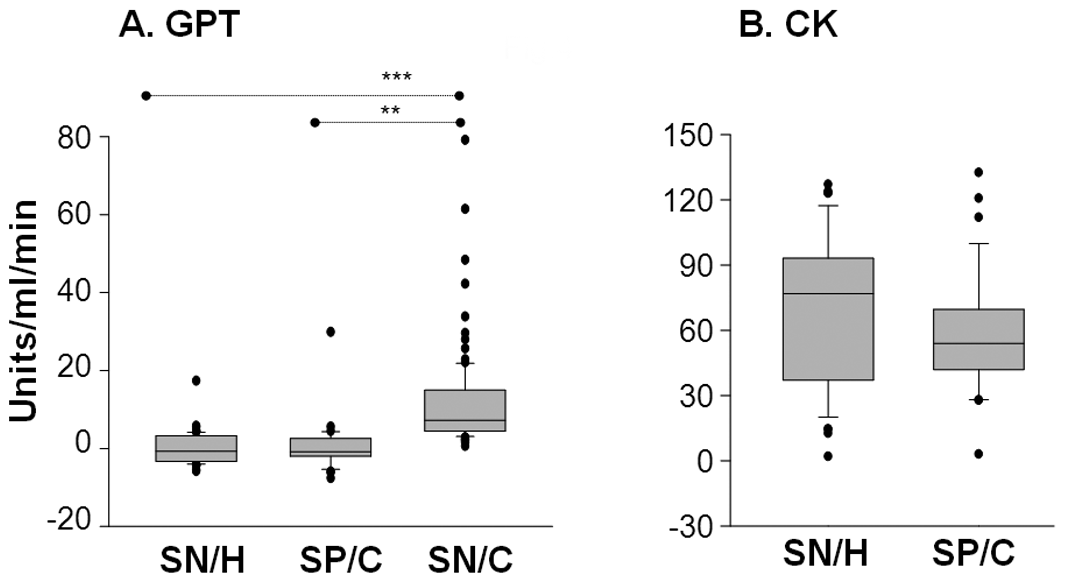
Metabolic markers of tissue injury were not significantly altered in chagasic subjects. Shown are plasma levels of activities of the glutamate pyruvate transaminase (**A**) and creatine kinase (**B**), determined by spectrophotometry.

CK is assayed as a marker of muscle damage in myocardial infarction. Increased serum level of GPT is often detected in congestive heart failure. We observed no significant increase in plasma and sera levels of CK and GPT activities in seropositive/chagasic subjects as compared to seronegative/healthy controls (Fig. 4). Seronegative/cardiac subjects exhibited a 3-fold increase in GPT activity when compared to that detected in normal/healthy controls or seropositive/chagasic subjects (Fig. 4A, p<0.01-0.001). These data suggest that traditional risk factors associated with heart failure are not good indicators of disease progression in seropositive/chagasic subjects.

### Stability of selected biomarkers and field relevance

We analyzed sera and plasma samples subjected to two cycles of freezing/thawing to determine if the above-studied biomarkers withstand temperature variance (Fig. S1). The observed increase in MPO activity and AOPP contents in freshly-frozen samples from seropositive subjects (Fig. 1B&C) were not detectable after freeze/thaw cycles (Fig. S1A&B). Nitrite level remained increased by 3-fold in seropositive/chagasic plasma subjected to freeze/thaw cycles (Fig. S1C, p<0.01), similar to that noted in freshly-frozen samples (Fig. 1E). The LPO and MDA levels, though decreased when compared to that noted in freshly-frozen samples (Fig. 2), remained increased by 6-fold and 2-fold, respectively, in seropositive plasma samples subjected to freeze-thaw cycles (Fig. S1D&E, p<0.001). Likewise, a significant decline of 70% and 80% in SOD activity and GSH contents, respectively, was detectable in seropositive/chagasic plasma samples subjected to freeze-thaw cycles (Fig. S1F&G, p<0.01). These data suggest that nitrite, LPO, MDA, SOD and GSH are stable metabolites, and can be employed to examine inflammatory/oxidative stress and antioxidant status in chagasic samples that may undergo temperature or storage irregularities or are collected at the field sites.

### Inter-relationship between selected biomarkers

**Figure 5:**
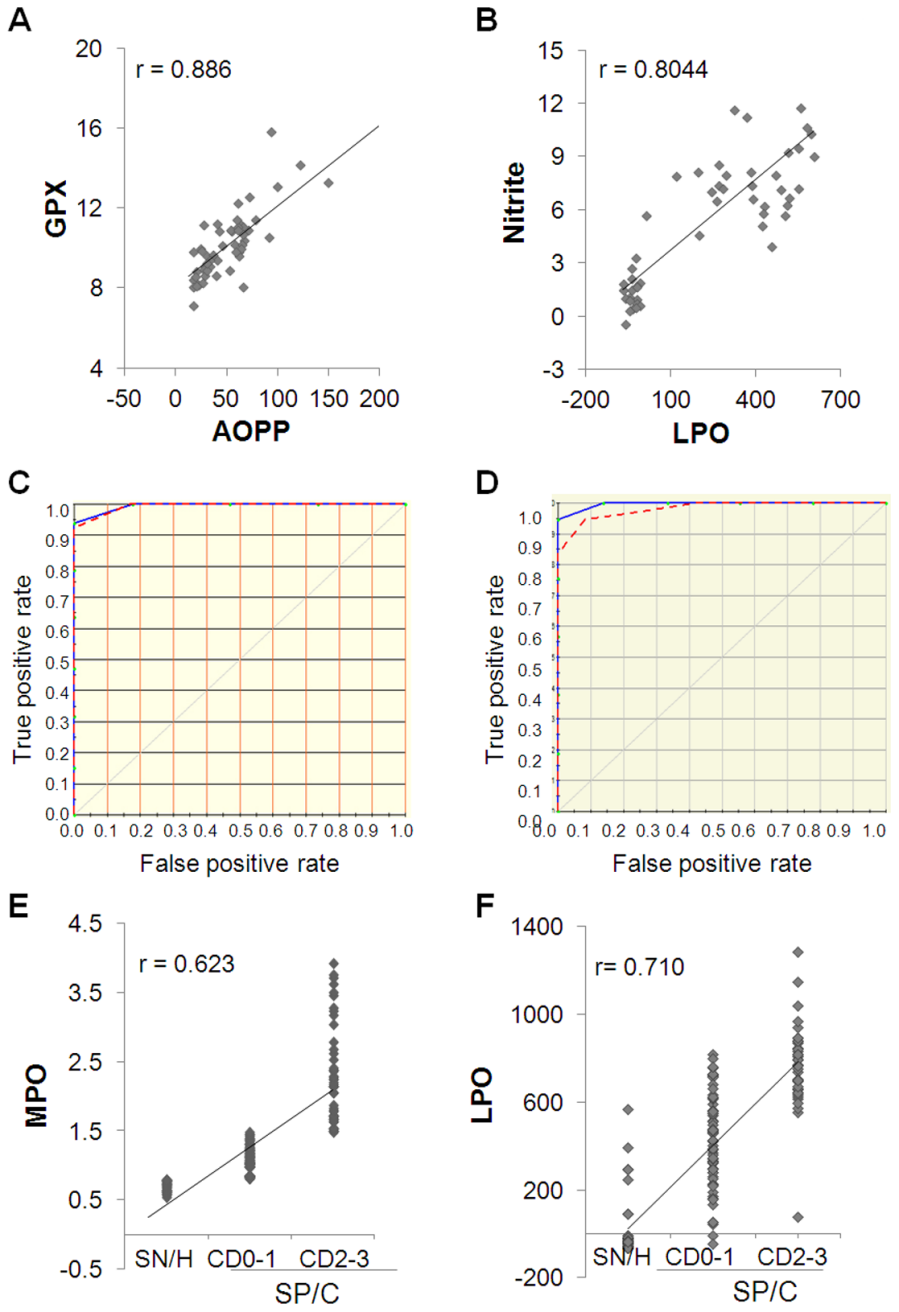
Pair-wise correlation and modeling analysis. Pair-wise correlation analysis of (**A**) nitrite (µmol/mg protein) with LPO (µmol/ml) and (**B**) glutathione peroxidase (units/mg protein) with AOPP (µmol/ml) utilizing data from plasma analysis of seropositive/chagasic and seronegative/healthy subjects is shown. (**C&D**) MARS analysis was performed using 80% of the data for various biomarkers from seronegative/healthy and seropositive/chagasic subjects as training dataset (blue curve) and 20% of the remaining data as test dataset to assess the performance of the model (red curve). Shown in panel C is MARS analysis of plasma levels of the biomarkers that revealed model fits perfectly (AUC/ROC = 1) on the training data for LPO, nitrite and SOD (with AUC/ROC of 0.099955 with testing dataset). Shown in panel D is MARS analysis of sera levels of the biomarkers that revealed model fits perfectly (AUC/ROC = 1) on the training data for MPO, LPO, and nitrite (with AUC/ROC of 0.9589 with testing dataset). (**E&F**) Shown are pair-wise correlation analyses of MPO (E) and LPO (F) contents with clinical disease. Each dot represents an individual subject.

Correlation analysis was performed to identify the strength of relationship between various biomarkers, and accepted as very strong with r value of >0.8 and moderately strong with r value of 0.6–0.8 (p<0.01, Table 2). Including data from seronegative/healthy and seropositive/chagasic subjects in the analysis, we observed a significant, moderately strong to very strong linear relationship between sera levels of MPO/AOPP, MPO/LPO, AOPP/nitrite, AOPP/MDA, nitrite/MDA, and LPO/MDA (all p<0.001, r≥0.600). Within the seropositive/chagasic group only, a strong relationship was observed for AOPP/nitrite (r = 0.877, p<0.001), AOPP/MDA (r = 0.711, p<0.001), MPO/SOD (r = 0.628, p<0.01) and AOPP/GSH (r = 0.806, p<0.001). When performing correlation analysis of plasma levels of various biomarkers in seropositive/chagasic subjects, we noted a significant, moderately strong linear relationship between MPO/AOPP, MPO/MDA, MPO/GPX, AOPP/MDA, AOPP/GPX, MDA/GPX, LPO/SOD, and GPX/GSH (all p<0.001, r≥0.600), the maximum strength being detected for AOPP/GPX (r = 0.886, p<0.001, Fig. 5A). A strong correlation between MPO/AOPP, AOPP/MDA and AOPP/GPX was maintained when plasma samples were subjected to freeze-thaw cycles (all p<0.001, r = 0.650–0.902). Correlation analysis of sera versus plasma levels of various parameters, including data from seronegative/healthy and seropositive/chagasic subjects, identified a very strong linear relationship between LPO and nitrite (r = 0.805, p<0.001, Fig. 5B) also. The observation of a strong correlation between increase in biomarkers of oxidative stress and inflammatory state, and between increase in oxidative stress (or inflammatory state) biomarkers and decrease in antioxidants suggest that inflammatory, oxidant and antioxidant responses are interlinked events in Chagas disease.

**Table 2:** Correlation analysis.

Parameters	Correlation coefficient (R value)	Significance (p value)	Correlation coefficient (R value)	Significance (p value)
	Including data from SN/H and SP/C subjects		Including data from SP/C subjects only	
**Sera/Sera**				
MPO/AOPP	0.664	0.000	0.608	0.004
MPO/LPO	0.600	0.000	-	-
MPO/SOD	-	-	0.628	0.003
AOPP/Nitrite	0.616	0.000	0.877	0.000
AOPP/MDA	0.634	0.000	0.711	0.000
AOPP/GSH	-	-	0.806	0.000
Nitrite/MDA	0.698	0.000	0.702	0.001
Nitrite/GSH	-	-	0.658	0.002
LPO/MDA	0.637	0.000	-	-
**Plasma/Plasma**				
MPO/AOPP	-	-	0.65	0.001
MPO/MDA	-	-	0.718	0.000
MPO/GPX	-	-	0.629	0.004
AOPP/MDA	-	-	0.802	0.000
AOPP/GPX	-	-	0.886	0.000
MDA/GPX	-	-	0.768	0.000
LPO/SOD	-	-	0.602	0.002
GPX/GSH	-	-	0.600	0.011
**Sera/Plasma**				
MPO/Nitrite	0.65	0.000	ND	ND
MDA/Nitrite	0.732	0.000	ND	ND
LPO/Nitrite	0.805	0.001	ND	ND
**Plasma/Plasma (subjected to freeze-thaw cycles)**				
MPO/AOPP	-	-	0.650	0.000
AOPP/MDA	-	-	0.802	0.000
AOPP/GPX	-	-	0.902	0.000
Nitrite/GPX	-	-	0.773	0.000

Plasma and sera samples from seronegative/healthy (SN/H, n = 45) and seropositive/chagasic (SP/C, n = 116) subjects were submitted to spectrophotometry analysis of various biomarkers of inflammation, oxidative stress, antioxidant status and cellular injury as described in Materials and Methods. Pearson's or Spearman's analysis was conducted to evaluate the strength of linear relationship between sera or plasma levels of biomarkers (among themselves) or with clinical disease category. Correlations coefficient (r) value of >0.8 was considered very strong and that of between 0.6–0.8 accepted as moderately strong [31]. “-”indicates a significant correlation at p<0.01 was not present. ND: not determined.

MARS analysis was performed to develop a classification model (Fig. 5C&D). Inputs to the model were the seronegative/healthy and seropositive/chagasic values for various parameters assessed in plasma and sera samples. To address the possible issue of over-fitting the data, we split the data into a training portion (80%) and a testing portion (20%). The blue curve in the model represents the fit of the model for the training dataset, i.e., 80% of the data that was utilized for creating the model; the 20% of the remaining data was used to assess the fit of the model for testing dataset (red curve). MARS modeling of the data collected from investigation of MPO, AOPP, nitrite, MDA, LPO, SOD and GSH in plasma is presented in Fig. 5C. The prediction success showed the model fits perfectly on the training data for LPO, nitrite and SOD (AUC/ROC value of 1.00, blue curve) and excellently on the testing dataset for LPO, nitrite and SOD (AUC/ROC value of 0.99955, red curve) (Fig. 5C). MARS modeling of the MPO, LPO, and nitrite levels in sera samples is shown in Fig. 5D. The prediction success showed the model fits perfectly on the training data (AUC/ROC value of 1.00, blue curve), and very well on the testing data (AUC/ROC value of 0.9589, red curve) for the three variables (Fig. 5D). These analyses suggest that plasma or sera levels of MPO, LPO, SOD and nitrite are highly specific and sensitive for distinguishing seropositive/chagasic subjects from seronegative/healthy controls, and the model developed based upon these variables will work well in predicting seropositive/chagasic patients from seronegative/healthy controls.

### Significance of selected biomarkers with respect to clinical disease state

Lastly, we determined if the studied parameters were associated with clinical disease status in seropositive/chagasic subjects. The Kruskal-Wallis/Dunn's analysis identified the plasma levels of MPO was significantly different between normal/healthy and CD0–CD1 stage chagasic patients (p<0.005), AOPP was significantly different between normal/healthy and CD2–CD3 stage chagasic patients (p<0.049), and nitrite, MDA, LPO, GSH, and SOD levels significantly distinguished the normal/healthy controls from CD0–CD1 or CD2–CD3 stages in chagasic subjects (all p<0.001). The Kruskal-Wallis/Dunn's analysis of the sera level of biomarkers with respect to disease category identified the AOPP and nitrite levels were significantly different between CD0–CD1 and CD2–CD3 stage chagasic patients (p<0.001), and MPO, MDA, and LPO levels significantly distinguished normal/healthy from CD0–CD1 and CD2–CD3 stage chagasic subjects (all p<0.001). None of the observed parameters indicated a significant difference between seronegative/healthy and seronegative/cardiac subjects.

Correlation analysis performed on the seronegative/healthy and seropositive/chagasic subjects suggested a significant, moderately strong linear relationship of clinical disease category with sera levels of MPO (r = 0.624, p<0.000, Fig. 5E) and LPO (r = 0.710, p<0.001, Fig. 5F), and fair correlation of clinical disease severity with sera levels of MDA (r = 0.512, p<0.001), AOPP (r = 0.436, p<0.001), and nitrite (r = 0.439, p<0.001). The plasma level of LPO also exhibited a moderately strong correlation with the clinical disease category (r = 0.625, p<0.001). MARS modeling with input of data for all variables and disease status from 80% of the seropositive/chagasic subjects showed the model fits on the training data with an ROC/AUC value of 0.786 for plasma level of LPO and ROC/AUC value of 0.696 for sera level of MPO. The 20% of the remaining datasets did not verify the sensitivity/specificity of the LPO- and MPO-based training model in distinguishing disease severity in seropositive/chagasic subjects. Together, these data suggest that selected biomarkers can significantly distinguish healthy controls from chagasic subjects; and MPO and LPO might be good indicators of clinical disease category in chagasic group.

## Discussion

**Figure 6:**
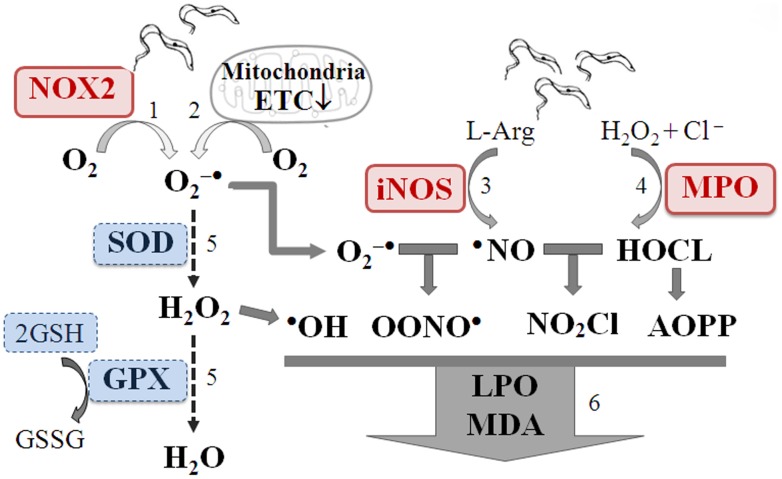
Inter-linked innate immune responses and oxidant/antioxidant status are major determinants of chronic Chagas disease. *Trypanosoma cruzi* or danger-associated molecular patterns (DAMPs) released due to cellular injuries stimulate ROS (O_2_^−•^, H_2_O_2_, ^•^OH) production by (1) NADPH oxidase–dependent oxidative burst in macrophages/monocytes and (2) mitochondrial membrane permeability transition and electron transport chain (ETC) inefficiency in non-phagocytes (e.g. cardiac myocytes). Likewise, *Tc* and DAMPs can activate phagocytes/neutrophils resulting in (3) iNOS-dependent ^•^NO and (4) myeloperoxidase-dependent HOCl production. If these reactive species are not scavenged by (5) activation of antioxidants (e.g. superoxide dismutases (SOD), glutathione peroxidase (GPX) and glutathione (GSH), then highly stable free radicals, e.g., peroxynitrite (^•^OONO), nitrosyl chloride and AOPPs are formed that (6) further inflict host cellular oxidative damage of lipids (e.g. LPO, MDA). The intracellular molecules (e.g., DNA, protein, lipids) that may be released from apoptotic or necrotic cells in oxidized form serve as DAMPs, sustaining the signaling activation of innate immune cells in chronically infected chagasic subjects.

In this study, we have investigated the selected biomarkers of innate immune response, and antioxidant/oxidant status in seropositive/chagasic and seronegative/cardiac subjects. Our data clearly show a significant increase in MPO, AOPP, nitrite (markers of innate immune response), LPO and MDA (markers of oxidative stress) and a decline in SOD and GSH (markers of antioxidant status) in seropositive/chagasic subjects. Seronegative/cardiac subjects exhibited a decline in SOD and a moderate increase in LPO and GPT levels; however, biomarkers of inflammation (MPO, AOPP, and nitrite) and MDA were not changed. Our data allow us to propose that oxidative/inflammatory stress is more pronounced in chagasic subjects with an infectious etiology, and LPO and MPO are potential biomarkers for identifying chagasic disease stage. To the best of our knowledge, this is the first report demonstrating the interlinked effects of innate responses, antioxidant status and oxidants levels in Chagas disease (Fig. 6).

Our data suggest that macrophages and neutrophils activation and formation of cytotoxic molecules (MPO, AOPP, and ^•^NO) persist in chronic *Tc* infection (Fig. 1). Our finding of a significant linear correlation between MPO and clinically-symptomatic disease provide the first evidence for the pathological significance of increased MPO activity in Chagas disease, and potential use of this biomarker in diagnosing disease severity. The stimuli that may drive the activation of innate immune cells in Chagas disease are not known. Considering the very low parasite burden in chronic stage, we believe that damage-associated molecular patterns (DAMPs) drive the activation of macrophages and neutrophils in chronic chagasic disease, to be determined in future studies.

A pro-oxidant milieu in seropositive humans has been demonstrated by increased GSSG/GSH ratio [25]–[26]. In this study, detection of 8–10-fold higher serum and plasma levels of LPO and MDA in seropositive/chagasic subjects, and the observation that LPO and MDA remained increased in seropositive samples subjected to long-term storage and freeze/thaw cycles demonstrate that these are stable markers for measuring oxidative stress in field-setting where immediate freezing of the freshly-collected samples may not be possible. A strong positive relationship between LPO levels and clinically-symptomatic disease suggest the prognostic value of LPO in identifying clinical disease in chagasic subjects, to be further verified using large-scale datasets in future studies. Multiple mechanisms are likely to contribute to increased LPO/MDA contents in chagasic subjects. One, increased sera/plasma levels of AOPP and ^•^NO in seropositive/chagasic subjects imply that the cytotoxic effects of free radicals released by innate immune cells would contribute to plasma LPO and MDA formation in chagasic subjects. Two, increase in plasma LPO/MDA levels in seropositive/chagasic subjects may also be due to oxidatively-modified lipids released as a consequence of cellular injuries incurred in the cardiac tissue. This notion is supported by the observation of intense oxidative modifications of DNA, lipids and proteins in chagasic myocardium [13], [15] and identification of multiple oxidatively-modified cardiac proteins in sera/plasma of chronically-infected animals [27]–[28] and humans [18]. Three, SOD and GPX/GSH antioxidants, utilized by mammalian cells to cope with free radicals, were compromised in chagasic myocardium [14], [17]. Decreased plasma levels of GPX in human chagasic subjects has been reported [29]. The observation of decreased SOD activity and GSH contents in plasma (Fig. 3) and peripheral blood cells of seropositive subjects [26] provide a strong evidence that human chagasic subjects are compromised in their capacity to activate antioxidant defense against oxidative stress. We surmise that increased plasma levels of MDA and LPO indicate that oxidant/antioxidant balance is tipped towards oxidative stress-induced damaging responses in chagasic subjects.

Serum is qualitatively and quantitatively different from plasma [30]. In serum, the bulk of the fibrinogen is removed by conversion into a fibrin clot together with the platelets which are either physically bound in the fibrin matrix or activated to form aggregates or both. During this process, varying amounts of other proteins are removed into the fibrin clot either by specific or non-specific interactions. Further, in the process of whole blood coagulation, the cellular elements (erythrocytes, leukocytes, platelets) can secrete components that are enriched in serum. For example, platelets contribute a variety of components to blood serum; e.g., vascular endothelial growth factor (VEGF) is detected at 250-pg/ml in serum and 30-pg/ml in plasma of healthy individuals [30]. Our observation of significant differences in plasma and serum levels of various biomarkers in this study support the notion that serum and plasma are not inter-changeable samples, and results derived from serum and plasma analysis should be carefully documented.

Seronegative/cardiac subjects in our study were categorized in NYHA functional class of II–III with NT-proBNP levels of >2000-ng/ml. However, except for a decline in plasma levels of SOD, and an increase in LPO and GPT levels, these subjects exhibited no change in other biomarkers that were altered in seropositive/chagasic subjects. We surmise that antioxidant/oxidant imbalance and cellular injury, though present, were not pronounced due to absence of inflammatory stress in seronegative/cardiac subjects.

Correlation coefficient (r) demonstrates the degree of linear relationship between the two variables. The r value of >0.8, 0.6–0.8, 0.3–0.5 and <0.3 are interpreted to exhibit a very strong, moderately strong, fair and poor linear relationships respectively [31]. Our data showed a very strong to moderately strong and significant (p<0.01) linear relationship between inflammatory markers (AOPP/Nitrate), inflammation and antioxidant status (AOPP/GPX, AOPP/GSH, Nitrite/GPX), and inflammation and oxidant status (Nitrite/LPO, MPO/TBARS) in seropositive/chagasic subjects. Of these, MPO, LPO and nitrite biomarkers were highly specific and sensitive for distinguishing seropositive/chagasic subjects from seronegative/healthy controls, evidenced by MARS modeling of the datasets. The AUC/ROC value of >0.95 of the testing dataset provides confidence that the model is not over-fitting the efficacy of MPO, LPO and nitrite in predicting seropositive/chagasic subjects. These data strongly suggest that innate immune cell responses and oxidant/antioxidant imbalance are interlinked and potential determinants of chronic Chagas disease. What might be the mechanisms linking oxidant/antioxidant imbalance and innate immune responses in chagasic disease are not known; however, the finding of neo-antigens (oxidized host molecules) as targets of antibody responses [32] and the ability of antibodies purified from the sera of chronically-infected individuals to trigger proliferative responses in PBMCs [33] provide clues. We propose that ROS of mitochondrial and inflammatory origin, coupled with an antioxidants' decline, leads to cellular oxidative damage. The intracellular molecules (e.g., DNA, protein, lipids) that may be released from apoptotic or necrotic cells are recognized by pattern recognition receptors, signaling activation of innate immune cells in chronically-infected chagasic subjects.

In summary, our data suggest interlinked effects of innate immune responses and oxidant/antioxidant imbalance play a major role in chronic phase of Chagas disease. This is evidenced by finding that increase in biomarkers of innate immune responses (MPO, AOPP, nitrite), oxidative stress (LPO, MDA) and a decline in antioxidant response (SOD, GSH, GPX) in seropositive/chagasic subjects was strongly correlated. We propose that a substantial effort should be made in delineating the signaling mechanisms contributing to complex interrelationship between oxidative stress and inflammatory mediators to identify the specific drug targets for controlling progressive chagasic cardiomyopathy. The finding of a significant correlation between the increase in MPO and LPO levels and clinical Chagas disease in this study provide us an impetus to test and verify the sensitivity and specificity of MPO and LPO in determining clinical Chagas disease in large-scale cross-sectional studies. If confirmed, these biomarkers will potentially be useful in designing predictive models for identifying the patients at the risk of developing the clinical disease.

## Supporting Information

Figure S1 **Impact of sample storage conditions on peripheral biomarkers of inflammation, oxidative stress and antioxidants.** Sera and plasma samples from seropositive and seronegative individuals were stored for >2 years and subjected to two cycle of freezing and thawing. The sera *(* ***A*** *)* and plasma *(* ***B–G*** *)* levels of MPO activity (**A**), AOPP contents (**B**), and nitrite levels (**C**) were determined as markers of neutrophil/phagocyte activation. The LPO (**D**) and MDA (**E**) contents were determined as biomarkers of oxidative stress. The SOD activity (**F**) and GSH content (**G**) were measured as indicators of antioxidant status. Data (mean of triplicate observations from each sample) are presented as box plot. The horizontal lines of the box (bottom to top) depict the lower quartile (Q1, cuts off lowest 25% of the data), median (Q2, middle value), and upper quartile (Q3, cuts off the highest 25% of the data). The lower and upper whiskers depict the smallest and largest non-outlier observations, respectively, and solid dots represent the outliers. The spacing between the different parts of the box indicates the degree of dispersion (spread). Standard deviation for triplicate observations for all samples was <12%.
(TIF)

Table S1 **Plasma and sera biomarkers in seropositive/chagasic subjects.**
(DOC)
