# Supplementary figures and images for: Innate Immune Responses and Antioxidant/Oxidant Imbalance Are Major Determinants of Human Chagas Disease
Source: PLoS Negl Trop Dis. 2013 Aug 8;7(8):e2364. doi: 10.1371/journal.pntd.0002364 (PMC3738450; doi:10.1371/journal.pntd.0002364)

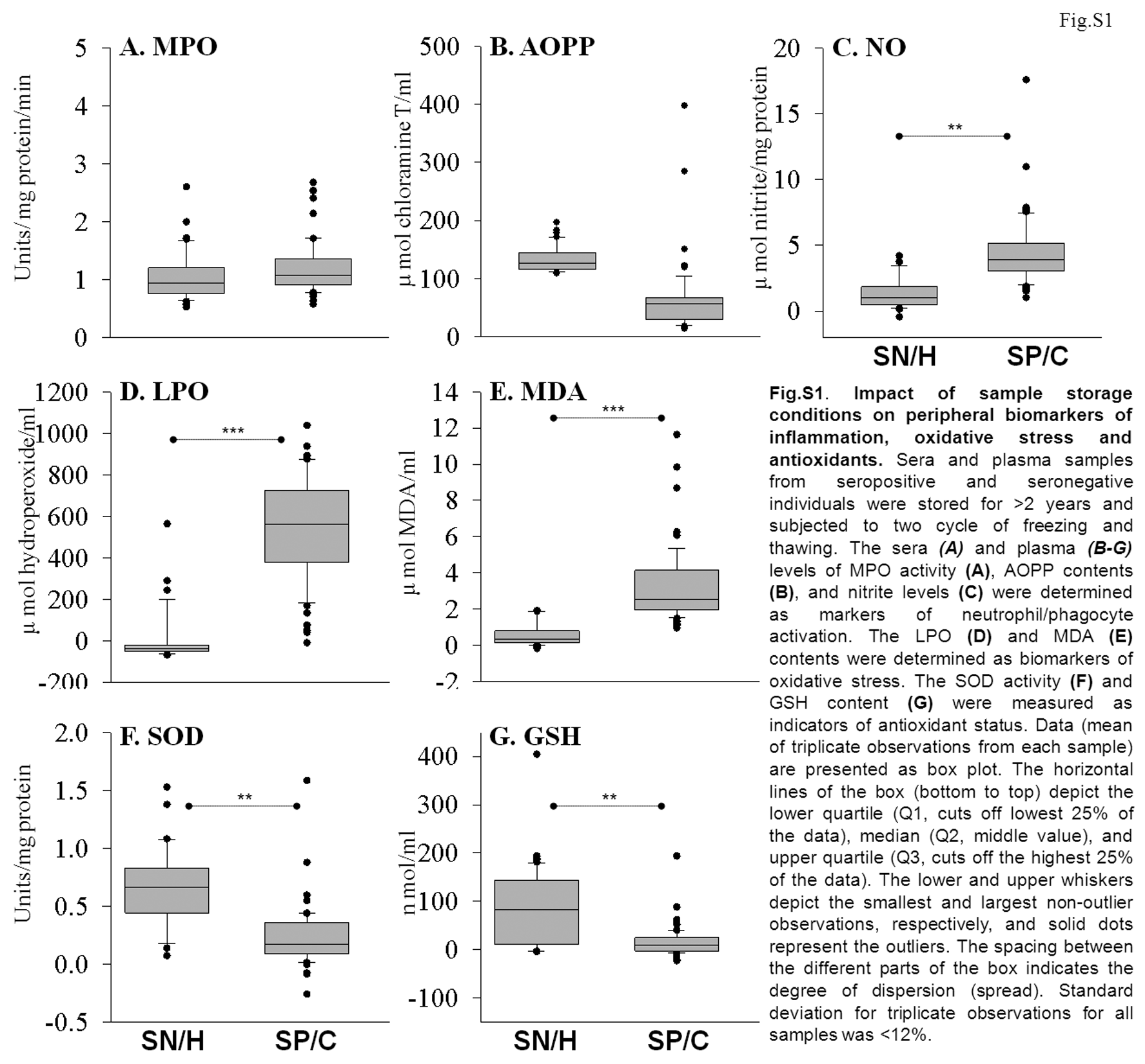

Supplement: Figure S1 — Impact of sample storage conditions on peripheral biomarkers of inflammation, oxidative stress and antioxidants. Sera and plasma samples from seropositive and seronegative individuals were stored for >2 years and subjected to two cycle of freezing and thawing. The sera ( A ) and plasma ( B–G ) levels of MPO activity (A), AOPP contents (B), and nitrite levels (C) were determined as markers of neutrophil/phagocyte activation. The LPO (D) and MDA (E) contents were determined as biomarkers of oxidative stress. The SOD activity (F) and GSH content (G) were measured as indicators of antioxidant status. Data (mean of triplicate observations from each sample) are presented as box plot. The horizontal lines of the box (bottom to top) depict the lower quartile (Q1, cuts off lowest 25% of the data), median (Q2, middle value), and upper quartile (Q3, cuts off the highest 25% of the data). The lower and upper whiskers depict the smallest and largest non-outlier observations, respectively, and solid dots represent the outliers. The spacing between the different parts of the box indicates the degree of dispersion (spread). Standard deviation for triplicate observations for all samples was <12%. (TIF) [file pntd.0002364.s001.tif]
