# Supplementary material for: Innate Immune Responses and Antioxidant/Oxidant Imbalance Are Major Determinants of Human Chagas Disease
Source: PLoS Negl Trop Dis. 2013 Aug 8;7(8):e2364. doi: 10.1371/journal.pntd.0002364 (PMC3738450; doi:10.1371/journal.pntd.0002364)
Supplement: Table S1 — Plasma and sera biomarkers in seropositive/chagasic subjects. (DOC) [file pntd.0002364.s002.doc]

| **Table S1: Plasma and sera biomarkers in seropositive/chagasic subjects** | | | | |  |
| --- | --- | --- | --- | --- | --- |
| **Parameter** | **Sample type** | **Mean ± SD (range)** | | | |
|  |  | **Seronegative/healthy** | **Seropositive/chagasic** | **Seronegative/cardiac** | |
| **Inflammatory markers** | | | | | |
| Myeloperoxidase  (units/mg protein/min) | Plasma | 0.99 ± 0.64 (0.64-0.89) | 1.32 ± 0.61 (0.99-3.44) | 1.00 ± 0.27 (0.58-2.9) | |
|  | Sera | 0.85 ± 0.21 (0.51-1.26) | 2.42 ± 0.1 (1.15-4.24) | ND | |
| AOPP  (µ mol chlor-amine-T/ml) | Plasma | 129.4 ± 19.37 (121-181) | 201.5 ± 20.9 (150-436) | 128.58 ± 56.3 (42.6-300.9) | |
|  | Sera | 182.01 ± 28.73 (130-194) | 231.5 ± 53.8 (173-392) | ND | |
| Nitrate/nitrite  (μ mol nitrite/mg protein) | Plasma | 1.39 ± 1.23 (1.34-4.73) | 8.05 ± 2.63 (3.94-18.7) | 1.54 ± 0.33(0.61-2.88) | |
|  | Sera | 3.30 ± 1.60 (0.95-6.9) | 4.61 ± 0.71 (2.15-17.33) | ND | |
| **Oxidative stress markers** | | | | | |
| Lipid peroxides  (µ mol hydro-peroxide/ml) | Plasma | 34.92 ± 10.53 (21.11-46.33) | 592.2 ± 24.60 (134-1282) | 171.30 ± 51.9 (94.7-392.9) | |
|  | Sera | 33.92 ± 14.89 (21-244) | 397.85 ± 11.29 (121-606) | ND | |
| Malondialdehydes  (µ mol TBARS/ml) | Plasma | 0.549 ± 0.24 (0.88-3.9) | 2.02 ± 0.2 (0.98-9.85) | 0.325 ± 0.09 (0.16-0.52) | |
|  | Sera | 0.62 ± 0.17 (1.34-3.45) | 3.53 ± 1.48 (1.91-9.99) | ND | |
| **Antioxidants** | | | | | |
| Superoxide dismutase  (units/mg protein) | Plasma | 0.687 ± 0.026 (0.277-0.76) | 0.32 ± 0.082 (0.003-0.54) | -0.014 ± 0.12 (-0.15-0.63) | |
|  | Sera | 0.527 ± 0.199 | 0.419 ± 0.202 | ND | |
| Glutathione peroxidase (units/mg protein) | Plasma | 9.71 ± 0.45 (8.98-10.52) | 10.39 ± 2.45 (7.84-17.82) | 9.40 ± 1.21 (5.2-11.46) | |
|  | Sera | 9.42 ± 2.46 | 8.843 ± 0.905 | ND | |
| Glutathione (n moles/ml) | Plasma | 85.07 ± 12.37 (136.8-99.87) | 21.64 ± 7.40 (3.84-404.43) | ND | |
|  | Sera | -2.196 ± 2.51 | -0.827 ± 2.61 | ND | |
| **Cellular injury markers** | | | | | |
| Glutamic pyruvic transaminase (units/ml/min) | Plasma | 3.27 ± 1.14 (0.57-5.81) | 3.72 ± 0.40 (0.76-5.62) | 12.69 ± 1.91 (1.40-79.19) | |
| Creatine kinase (units/ml/min) | Plasma | 60.13 ± 34.0 (1.90-127.0) | 58.44 ± 26.55 (3.08-132.0) | ND | |

Plasma and sera samples from seronegative/healthy (n=45), seropositive/chagasic (n=116) and seronegative/cardiac (n=102) subjects were submitted to spectrophotometric analysis of various biomarkers of inflammation, oxidative stress, antioxidant status and cellular injury as described in Materials and Methods. Data are presented as mean ± standard deviation (range of values obtained from analysis of all subjects within the group).
